# Supplementary material for: First-line systemic treatment strategies for unresectable hepatocellular carcinoma: A cost-effectiveness analysis
Source: PLoS One. 2023 Apr 13;18(4):e0279786. doi: 10.1371/journal.pone.0279786 (PMC10101629; doi:10.1371/journal.pone.0279786)

**Supporting information**

S1 Fig. One-way sensitivity analyses of atezolizumab plus bevacizumab (A), brivanib (B), linifanib (C), sorafenib plus erlotinib (D), donafenib(E), lenvatinib(F), sintilimab plus IBI305(G), sunitinib(H) in comparison with sorafenib, linifanib(I), donafenib(J), sorafenib plus erlotinib(K) in comparison with lenvatinib, sintilimab plus IBI305 vs. atezolizumab plus bevacizumab(M)

A:


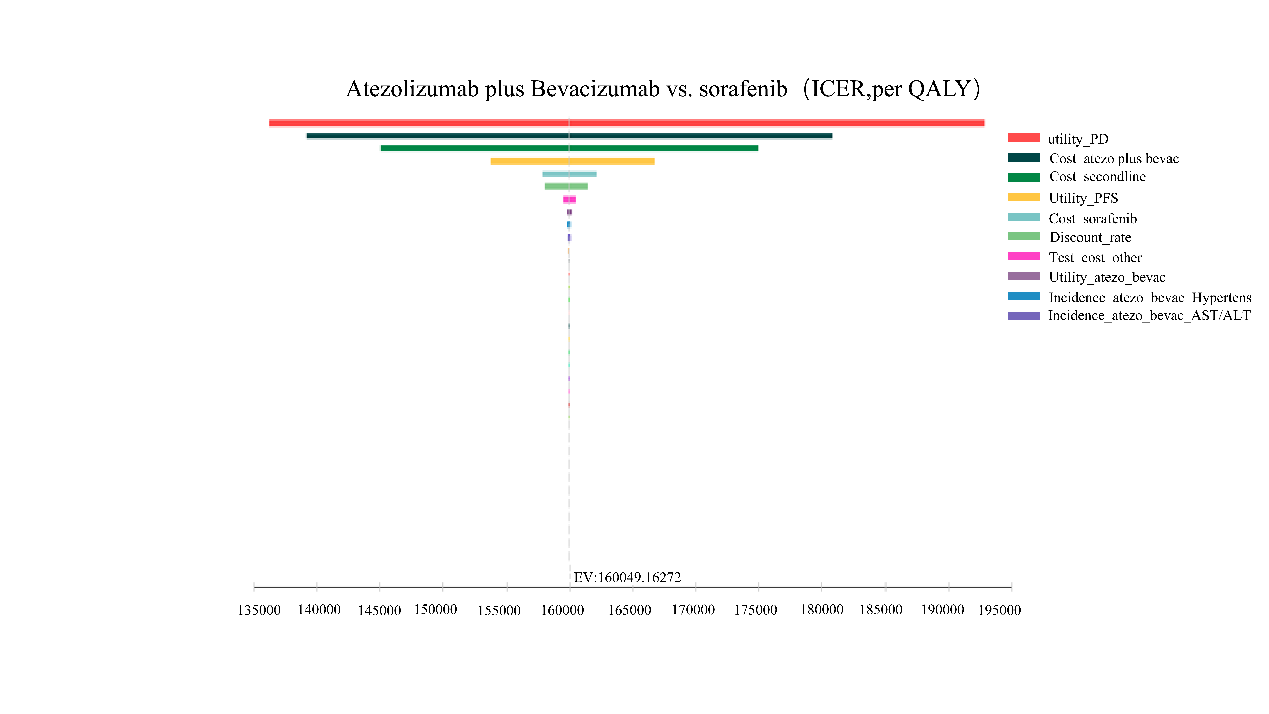


B:


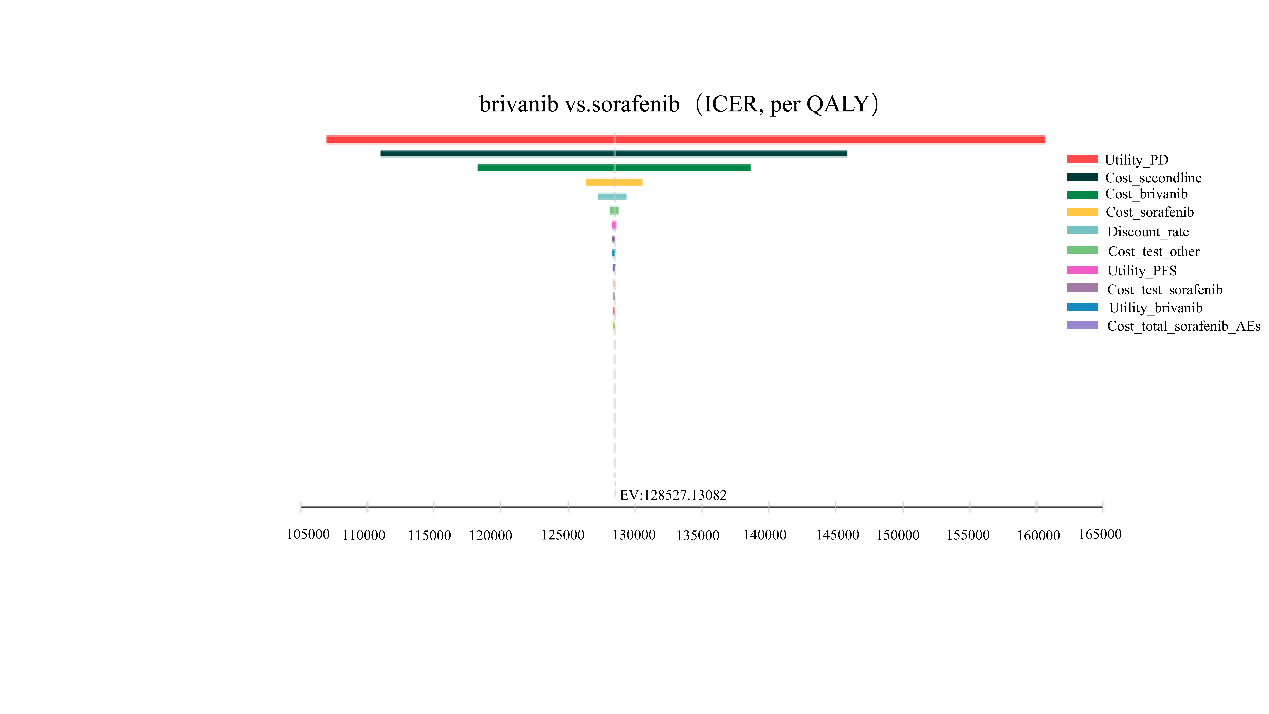


C:


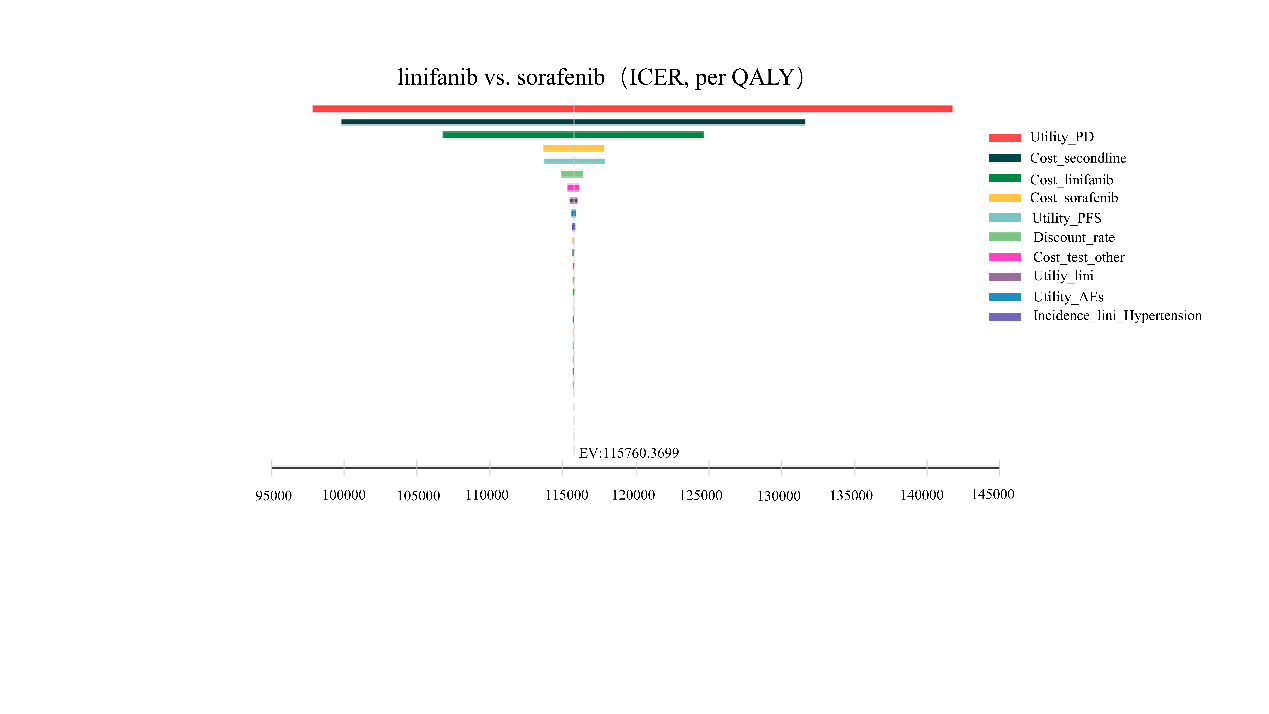


D:


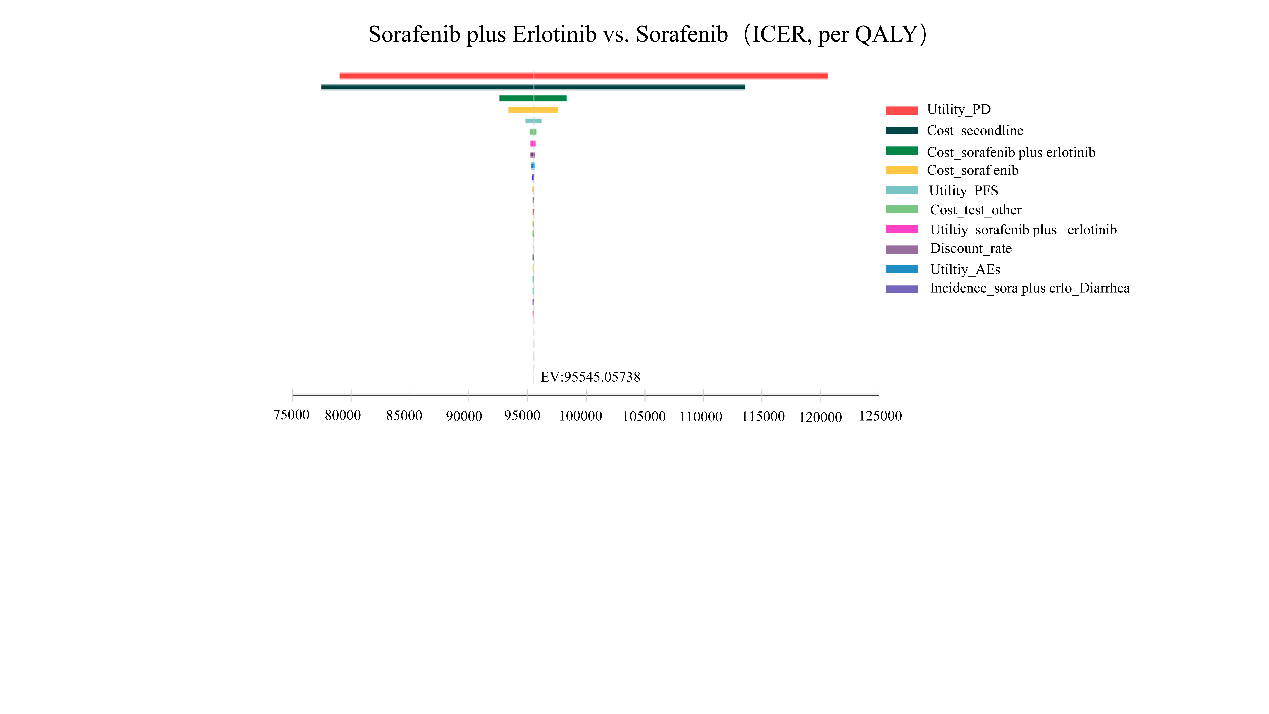


E:


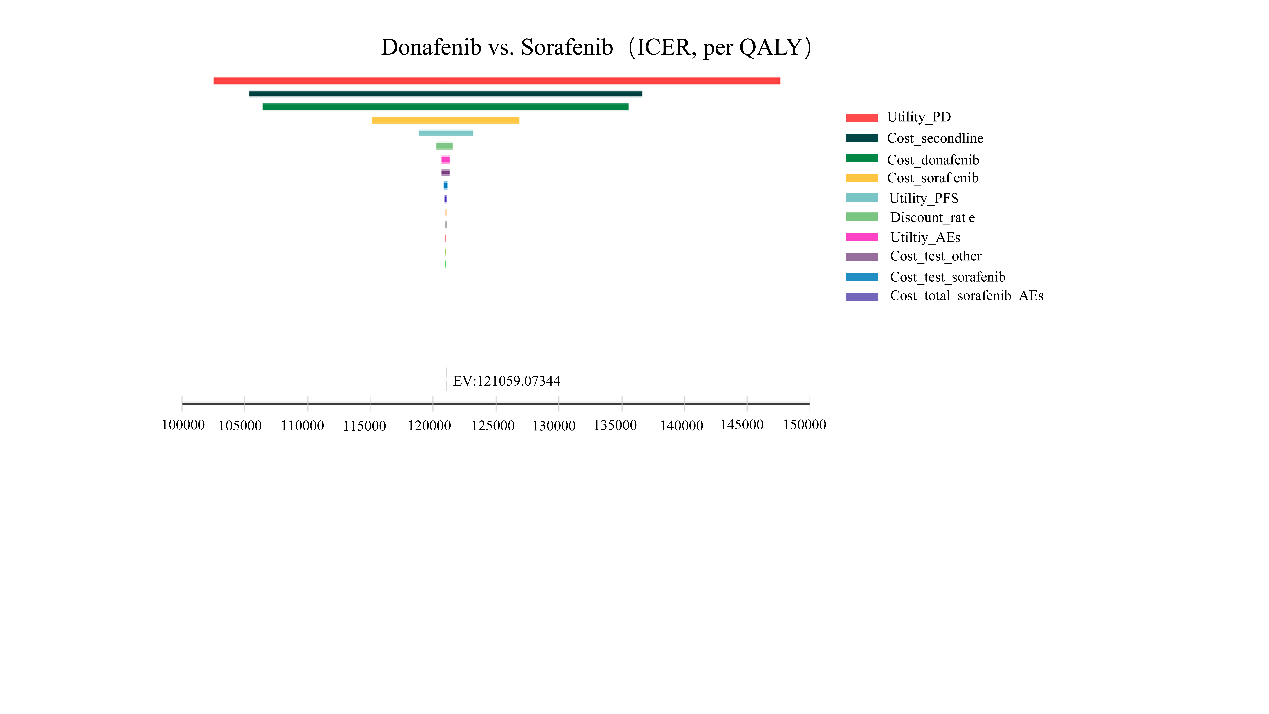


F:


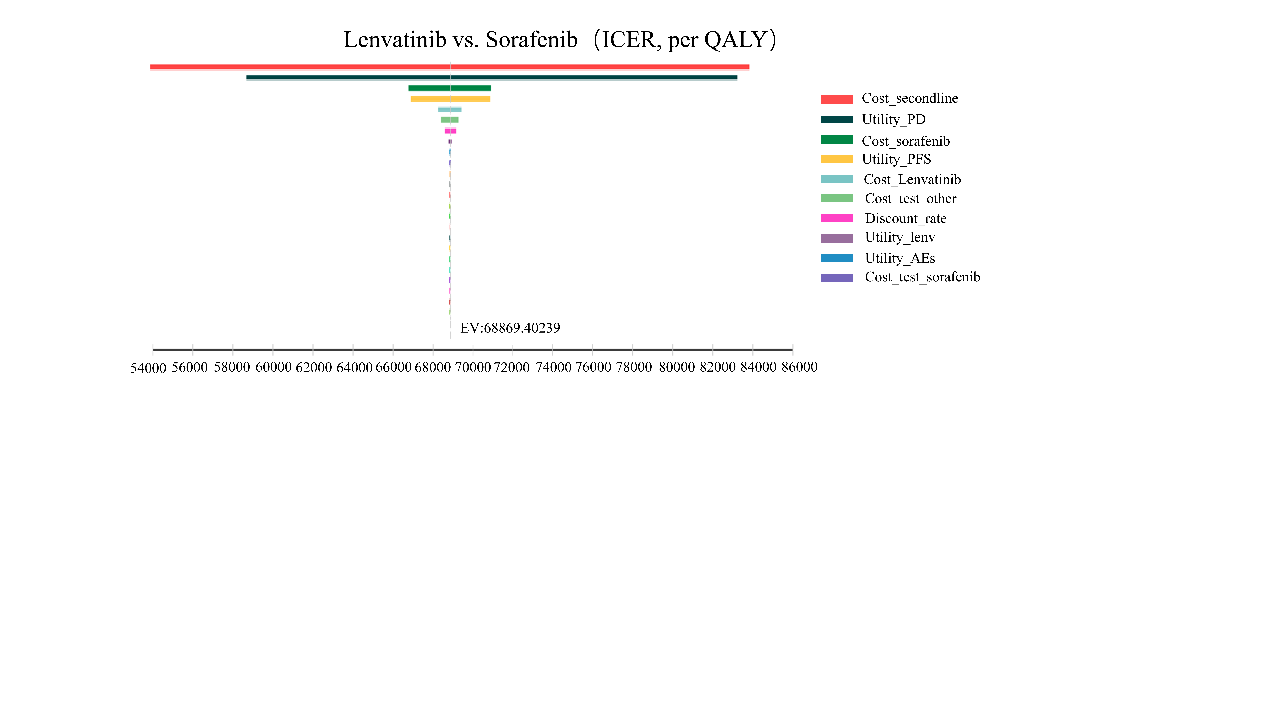


G:


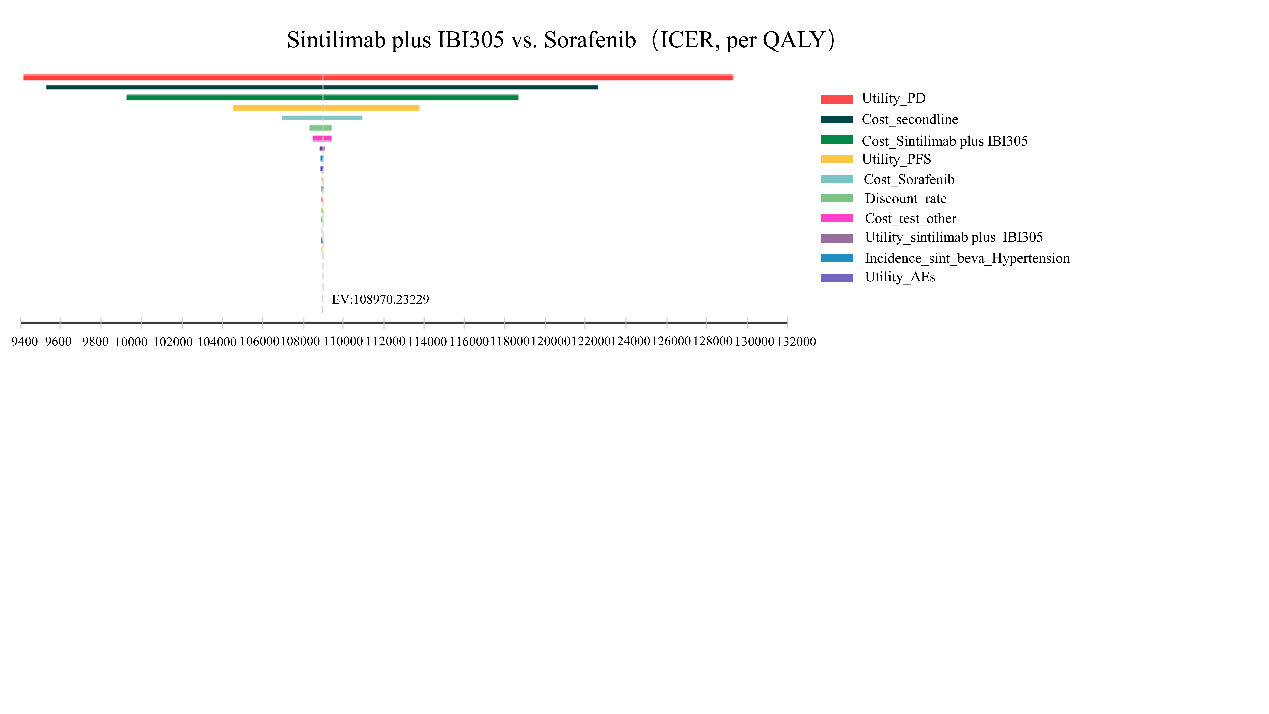


H:


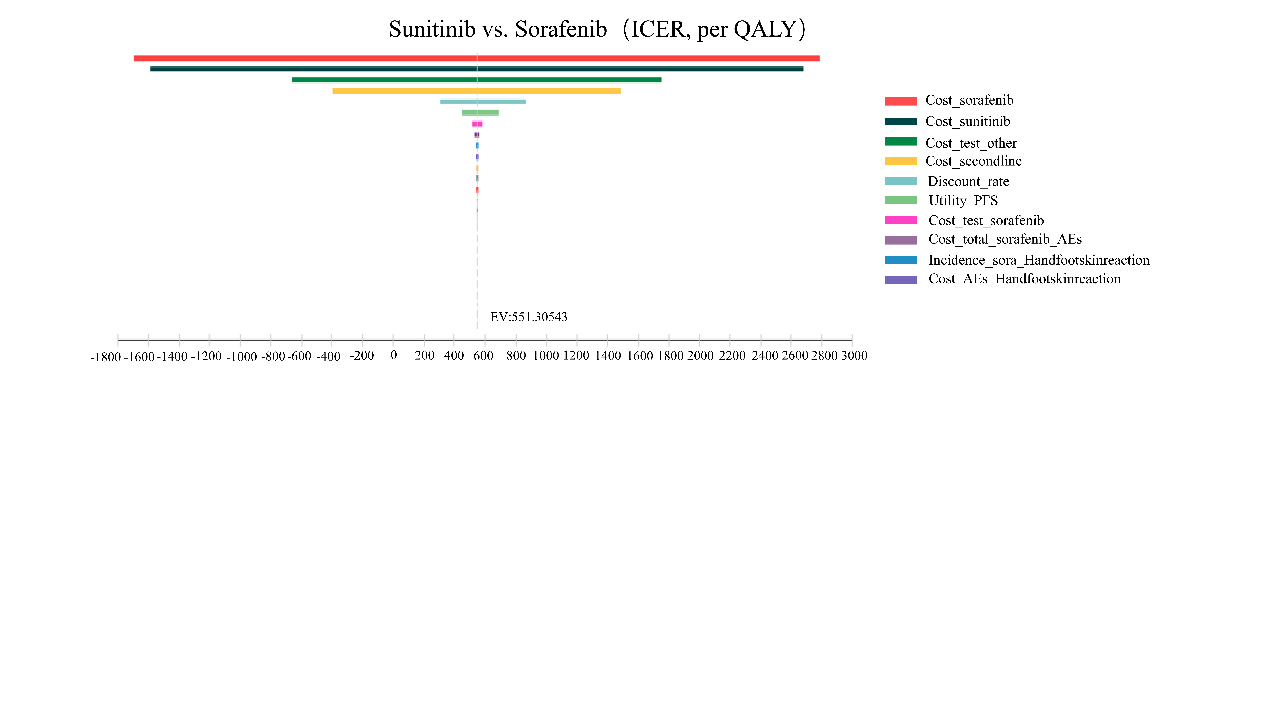


I:


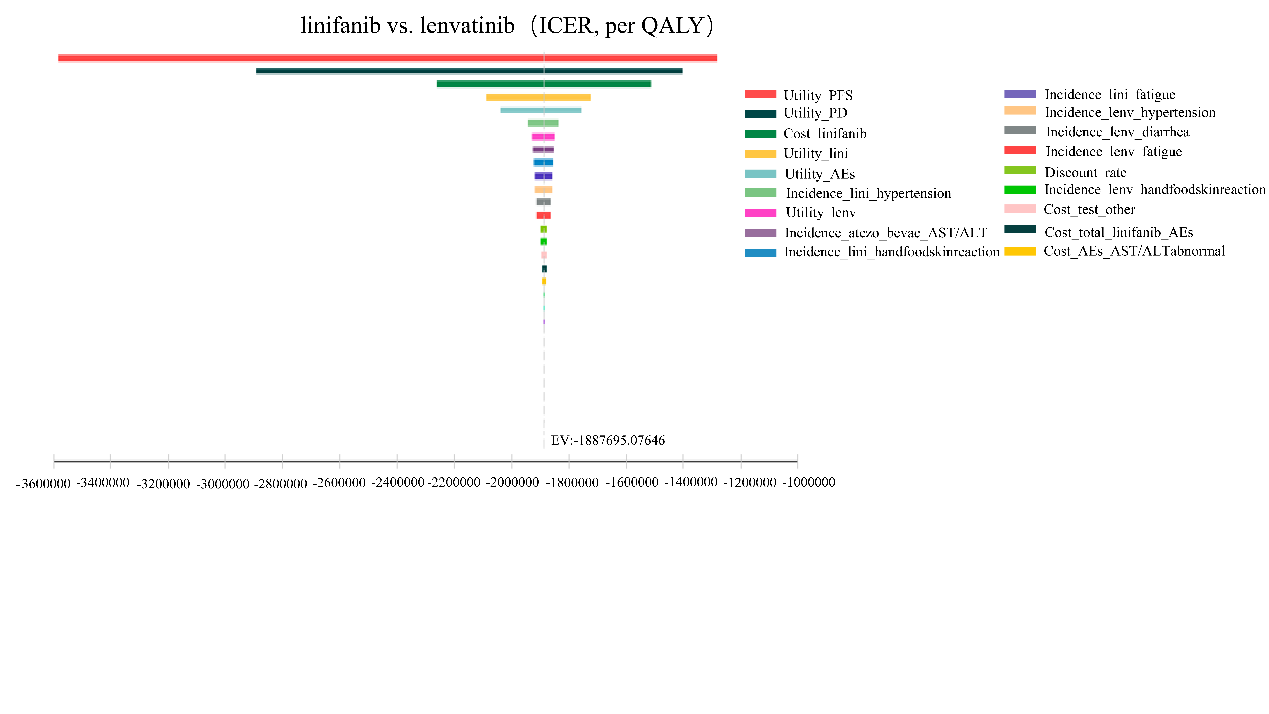


J:


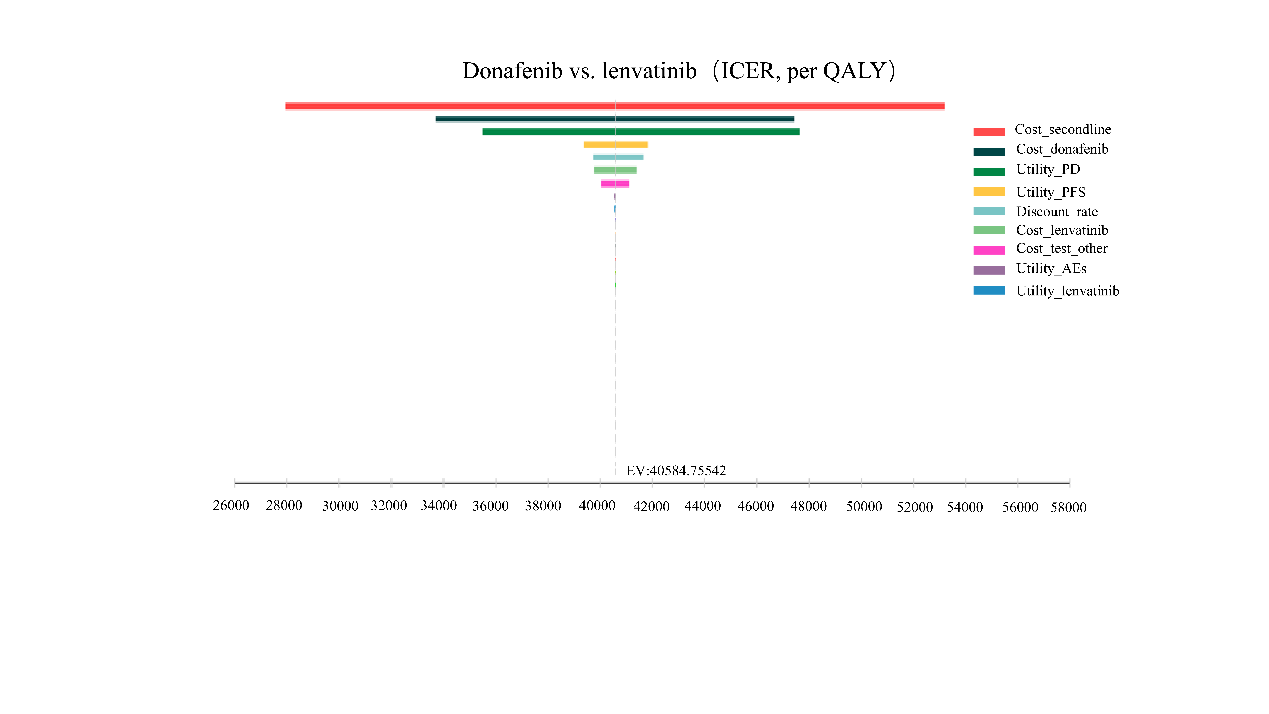


K:


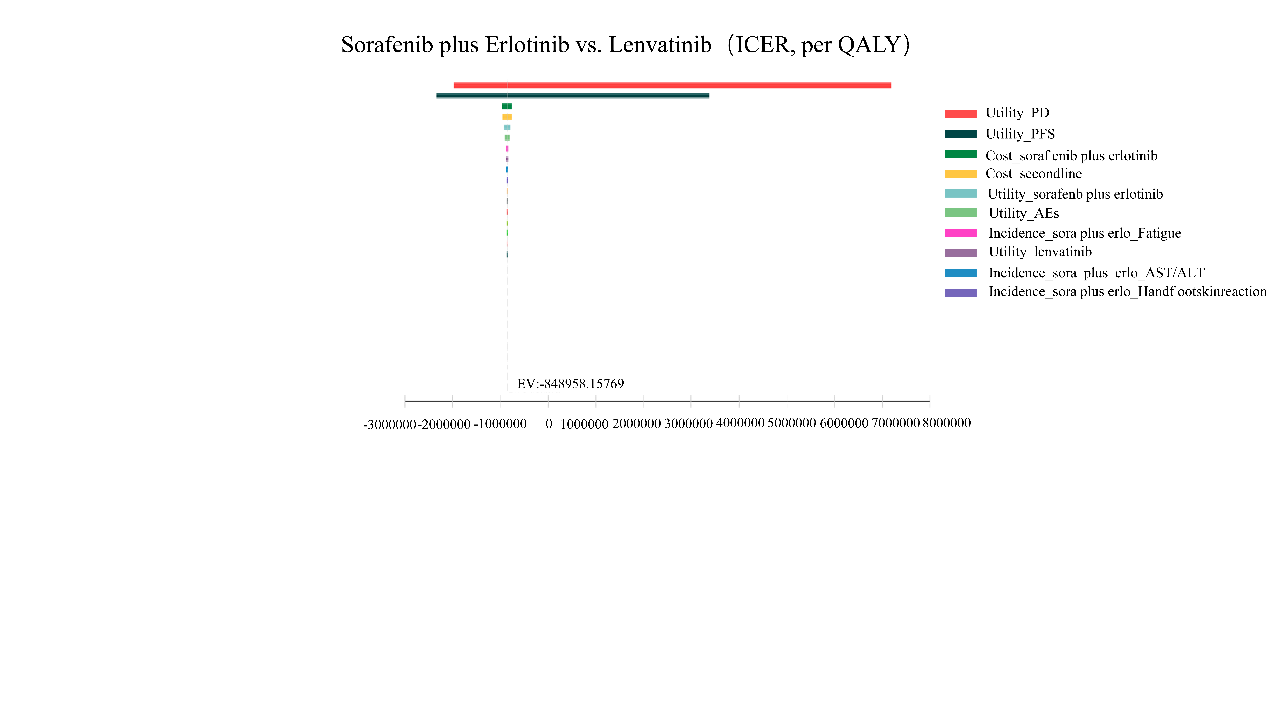


L:


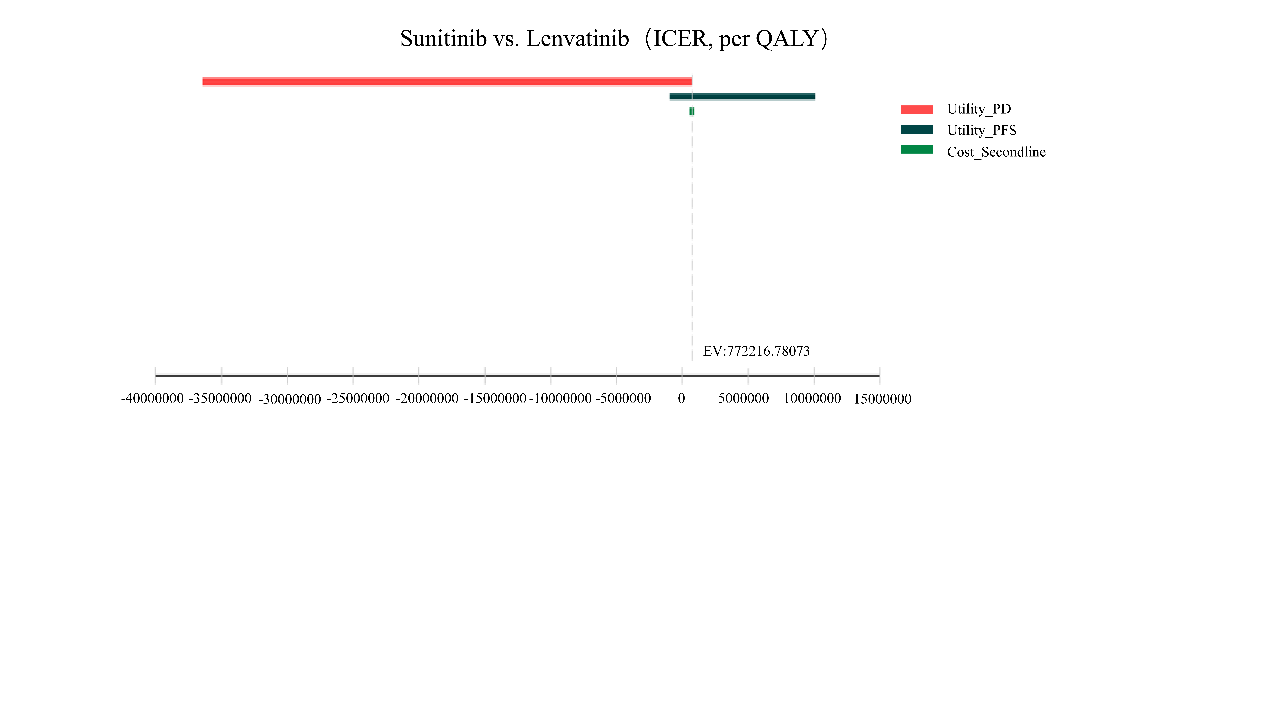


M:


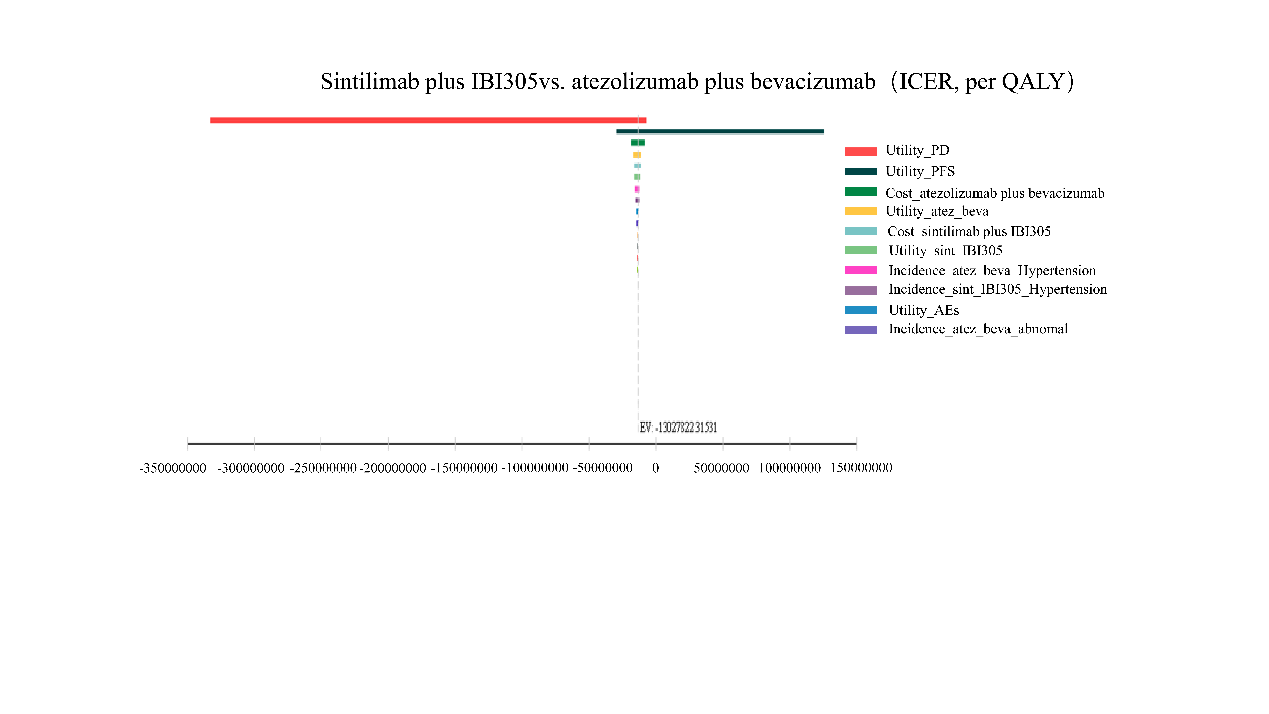

Supplement: S1 Fig — One-way sensitivity analyses of atezolizumab plus bevacizumab (A), brivanib (B), linifanib (C), sorafenib plus erlotinib (D), donafenib(E), lenvatinib(F), sintilimab plus IBI305(G), sunitinib(H) in comparison with sorafenib, linifanib(I), donafenib(J), sorafenib plus erlotinib(K) in comparison with lenvatinib, sintilimab plus IBI305 vs. atezolizumab plus bevacizumab(M). (DOCX) [file pone.0279786.s001.docx]
