# Supplementary material for: First-line systemic treatment strategies for unresectable hepatocellular carcinoma: A cost-effectiveness analysis
Source: PLoS One. 2023 Apr 13;18(4):e0279786. doi: 10.1371/journal.pone.0279786 (PMC10101629; doi:10.1371/journal.pone.0279786)
Supplement: S1 Table — (DOCX) [file pone.0279786.s002.docx]

**Supporting information**

S1 Table The original data of the network meta

| **Certainty assessment** | | | | | | | | | | **№ of patients** | | **Effect** | | **Certainty** | **Importance** | |
| --- | --- | --- | --- | --- | --- | --- | --- | --- | --- | --- | --- | --- | --- | --- | --- | --- |
| **№ of studies** | **Study design** | | | | **Risk of bias** | **Inconsistency** | **Indirectness** | **Imprecision** | **Other considerations** | **First line**  **OS** | **Risk of**  **Death**  **in**  **Control** | **Relative**  **(95% CI)** | **Absolute**  **(95% CI)** |  |  |  |
| **Atezolizumab + Bevacizumab vs Sorafenib - Overall survival** | | | | | | | | | | | | | | | | |
| 1 | randomised trials | | | | not serious | not serious | not serious | not serious | none |  |  | **HR 0.58** **(0.42 to 0.79)** |  | ⨁⨁⨁⨁ High | CRITICAL | |
| **Donafenib vs Sorafenib - Overall survival** | | | | | | | | | | | | | | | | |
| 1 | randomised trials | | | | not serious | not serious | not serious | not serious | none |  |  | **HR 0.83** **(0.69 to 0.98)** |  | ⨁⨁⨁⨁ High | CRITICAL | |
| **Lenvatinib vs Sorafenib - Overall survival** | | | | | | | | | | | | | | | | |
| 1 | randomised trials | | | | not serious | not serious | not serious | serious | none |  |  | **HR 0.92** **(0.79 to 1.06)** |  | ⨁⨁⨁◯ Moderate | CRITICAL | |
| **Linifanib vs Sorafenib - Overall survival** | | | | | | | | | | | | | | | | |
| 1 | | randomised trials | | | not serious | not serious | not serious | serious | none |  |  | **HR 1.05 (0.90 to 1.22)** |  | ⨁⨁⨁◯ Moderate | CRITICAL | |
| **Sintilimab + Bevacizumab vs Sorafenib - Overall survival** | | | | | | | | | | | | | | | | |
| 1 | | | randomised trials | | not serious | not serious | not serious | not serious | none |  |  | **HR 0.57 (0.43 to 0.75)** |  | ⨁⨁⨁⨁ High | CRITICAL | |
| **Sorafenib vs Brivanib - Overall survival** | | | | | | | | | | | | | | | | |
| 2 | | | | randomised trials | not serious | not serious | not serious | serious | none |  |  | **HR 1.07 (0.94 to 1.23)** |  | ⨁⨁⨁◯ Moderate | CRITICAL | |
| **Sorafenib vs Placebo - Overall survival** | | | | | | | | | | | | | | | | |
| 1 | randomised trials | | | | not serious | not serious | not serious | not serious | none |  |  | **HR 0.65 (0.53 to 0.78)** |  | ⨁⨁⨁⨁ High | | CRITICAL |
| **Sunitinib vs Sorafenib - Overall survival** | | | | | | | | | | | | | | | | |
| 1 | randomised trials | | | | not serious | not serious | not serious | not serious | none |  |  | **HR 1.30 (1.13 to 1.5)** |  | ⨁⨁⨁⨁ High | | CRITICAL |
| **Atezolizumab + Bevacizumab vs Sorafenib - Progression free survival** | | | | | | | | | | | | | | | | |
| 1 | randomised trials | | | | not serious | not serious | not serious | not serious | none |  |  | **HR 0.59(0.47 to 0.76)** |  | randomised trials | | not serious |
| **Donafenib vs Sorafenib - Progression free survival** | | | | | | | | | | | | | | | | |
| 1 | randomised trials | | | | not serious | not serious | not serious | serious | none |  |  | **HR 0.91(0.76 to 1.08)** |  | randomised trials | | not serious |
| **Lenvatinib vs Sorafenib - Progression free survival** | | | | | | | | | | | | | | | | |
| 1 | randomised trials | | | | not serious | not serious | not serious | not serious | none |  |  | **HR 0.66(0.57 to 0.77)** |  | randomised trials | | not serious |
| **Linifanib vs Sorafenib - Progression free survival** | | | | | | | | | | | | | | | | |
| 1 | randomised trials | | | | not serious | not serious | not serious | not serious | none |  |  | **HR 0.76(0.64 to 0.90)** |  | randomised trials | | not serious |
| **Sintilimab + Bevacizumab vs Sorafenib - Progression free survival** | | | | | | | | | | | | | | | | |
| 1 | randomised trials | | | | not serious | not serious | not serious | not serious | none |  |  | **HR 0.56(0.46 to 0.70)** |  | randomised trials | | not serious |
| **Sorafenib vs Brivanib - Progression free survival** | | | | | | | | | | | | | | | | |
| 1 | randomised trials | | | | not serious | not serious | not serious | serious | none |  |  | **HR 1.01(0.88 to 1.16)** |  | randomised trials | | not serious |
| **Sorafenib vs Placebo - Progression free survival** | | | | | | | | | | | | | | | | |
| 2 | randomised trials | | | | not serious | not serious | not serious | serious | none |  |  | **HR 0.95(0.81 to 1.12)** |  | randomised trials | | not serious |
| **Sunitinib vs Sorafenib - Progression free survival** | | | | | | | | | | | | | | | | |
| 1 | randomised trials | | | | not serious | not serious | not serious | serious | none |  |  | **HR 1.13（0.99-1.30）** |  | randomised trials | | not serious |
