# Supplementary material for: First-line systemic treatment strategies for unresectable hepatocellular carcinoma: A cost-effectiveness analysis
Source: PLoS One. 2023 Apr 13;18(4):e0279786. doi: 10.1371/journal.pone.0279786 (PMC10101629; doi:10.1371/journal.pone.0279786)
Supplement: S2 Table — (DOCX) [file pone.0279786.s003.docx]

**Supporting information**

S2 Table: Key Model Inputs.

| Parameter | Expected value (range) | Distribution | References |
| --- | --- | --- | --- |
| Clinical input |  |  |  |
| Survival model for sorafenib |  |  |  |
| Log-logistic model for OS | gamm=1.381  lamb=0.091 |  | 5, 6 |
| Log-logistic model for PFS | gamma=2.134 lamb=0.348 |  | 5, 6 |
| HR for OS associated with Sunitinib vs. sorafenib | 1.30(1.13-1.5) | Log-normal | 16 |
| HR for PFS associated with Sunitinib vs. sorafenib | 1.13(0.99-1.30) | Log-normal | 16 |
| HR for OS associated with Brivanib vs. sorafenib | 1.07(0.94-1.23) | Log-normal | 16 |
| HR for PFS associated with Brivanib vs. sorafenib | 1.01(0.88-1.16) | Log-normal | 16 |
| HR for OS associated with Linifanib vs. sorafenib | 1.05(0.90-1.22) | Log-normal | 16 |
| HR for PFS associated with Linifanib vs. sorafenib | 0.76(0.64-0.90) | Log-normal | 16 |
| HR for OS associated with Sorafenib Plus Erlotinib vs. sorafenib | 0.93(0.78-1.11) | Log-normal | 16 |
| HR for PFS associated with Sorafenib Plus Erlotinib vs. sorafenib | 1.14(0.94-1.37) | Log-normal | 16 |
| HR for OS associated with Lenvatinib vs. sorafenib | 0.92(0.79-1.06) | Log-normal | 16 |
| HR for PFS associated with Lenvatinib vs. sorafenib | 0.66(0.57-0.77) | Log-normal | 16 |
| HR for OS associated with Atezolizumab plus Bevacizumab vs. sorafenib | 0.58(0.42-0.79) | Log-normal | 16 |
| HR for PFS associated with Atezolizumab plus Bevacizumab vs. sorafenib | 0.59(0.47-0.76) | Log-normal | 16 |
| HR for OS associated with Donafenib vs. sorafenib | 0.83(0.69-0.98) | Log-normal | 16 |
| HR for PFS associated with Donafenib vs. sorafenib | 0.91(0.76-1.08) | Log-normal | 16 |
| HR for OS associated with sintilimab-bevacizumab biosimilar vs. sorafenib | 0.57(0.43-0.75) | Log-normal | 16 |
| HR for PFS associated with sintilimab-bevacizumab biosimilar vs. sorafenib | 0.56(0.46-0.70) | Log-normal | 16 |
| Sorafenib: risk of AEs |  |  |  |
| Diarrhea | 0.066(0.0528-0.079) | Beta | 5, 6 |
| Hand-foot skin reaction | 0.119(0.095-0.143) | Beta | 5, 6 |
| Fatigue | 0.050(0.04-0.06) | Beta | 5, 6 |
| Hypertension | 0.078(0.062-0.094) | Beta | 5, 6 |
| AST/ALT abnormal | 0.053(0.042-0.064) | Beta | 5, 6 |
| Sunitinib: risk of AEs |  |  |  |
| Diarrhea | 0.072(0.058-0.087) | Beta | 8 |
| Hand-foot skin reaction | 0.133(0.106-0.160) | Beta | 8 |
| Fatigue | 0.063(0.050-0.075) | Beta | 8 |
| Hypertension | 0.038(0.030-0.046) | Beta | 8 |
| AST/ALT abnormal | 0 | / | 8 |
| Brivanib: risk of AEs |  |  |  |
| Diarrhea | 0.012(0.010-0.015) | Beta | 9 |
| Hand-foot skin reaction | 0.026(0.021-0.031) | Beta | 9 |
| Fatigue | 0.012(0.010-0.015) | Beta | 9 |
| Hypertension | 0.016(0.013-0.019) | Beta | 9 |
| AST/ALT abnormal | 0 | / | 9 |
| Sorafenib Plus Erlotinib:risk of AEs |  |  |  |
| Diarrhea | 0.251 | Beta | 10 |
| Hand-foot skin reaction | 0.102(0.082-0.123) | Beta | 10 |
| Fatigue | 0.177(0.141-0.212) | Beta | 10 |
| Hypertension | 0.047(0.038-0.056) | Beta | 10 |
| AST/ALT abnormal | 0.138(0.110-0.166) | Beta | 10 |
| Linifanib: risk of AEs |  |  |  |
| Diarrhea | 0.120(0.077-0.144) | Beta | 11 |
| Hand-foot skin reaction | 0.137(0.110-0.165) | Beta | 11 |
| Fatigue | 0.096(0.077-0.115) | Beta | 11 |
| Hypertension | 0.208(0.166-0.250) | Beta | 11 |
| AST/ALT abnormal | 0.143(0.042-0.063) | Beta | 11 |
| Lenvatinib: risk of AEs |  |  |  |
| Diarrhea | 0.042(0.034-0.050) | Beta | 12 |
| Hand-foot skin reaction | 0.029(0.024-0.035) | Beta | 12 |
| Fatigue | 0.038(0.030-0.045) | Beta | 12 |
| Hypertension | 0.233(0.038-0.056) | Beta | 12 |
| AST/ALT abnormal | 0 | / | 12 |
| Atezolizumab plus Bevacizumab: risk of AEs |  |  |  |
| Diarrhea | 0.018(0.015-0.022) | Beta | 13 |
| Hand-foot skin reaction | 0 | / | 13 |
| Fatigue | 0.024(0.020-0.029) | Beta | 13 |
| Hypertension | 0.152(0.122-0.182) | Beta | 13 |
| AST/ALT abnormal | 0.07(0.014-0.022) | Beta | 13 |
| Donafenib: risk of AEs |  |  |  |
| Diarrhea | 0.018(0.014-0.022) | Beta | 14 |
| Hand-foot skin reaction | 0.057(0.046-0.069) | Beta | 14 |
| Fatigue | 0 | / | 14 |
| Hypertension | 0.090(0.072-0.108) | Beta | 14 |
| AST/ALT abnormal | 0.018(0.014-0.022) | Beta | 14 |
| Sintilimab–bevacizumab IBI305: risk of AEs |  | Beta |  |
| Diarrhea | 0.016(0.013- 0.019) | Beta | 15 |
| Hand-foot skin reaction | 0 | / | 15 |
| Fatigue | 0 | / | 15 |
| Hypertension | 0.145(0.116- 0.174) | Beta | 15 |
| AST/ALT abnormal | 0.032(0.025- 0.038) | Beta | 15 |
| Utility input |  |  |  |
| Utility of PFS | 0.760(0.608- 0.912) | Beta | 27 |
| Utility of PD | 0.680(0.544- 0.816) | Beta | 27 |
| Disutility due to AEs |  |  |  |
| Grade 3 and higher | 0.160(0.128- 0.192) | Beta | 28 |
| AEs cost, $/event |  |  |  |
| Diarrhea | 3.290(2.632- 3.95) | Gamma | 27 |
| Hand-foot skin reaction | 33.640(26.912- 40.368) | Gamma | 27 |
| Fatigue | 3.012(2.410- 3.614) | Gamma | 30 |
| Hypertension | 1.355(1.084-1.626) | Gamma | 28 |
| AST/ALT abnormal | 59.236(47.390- 71.080) | Gamma | 30 |
| Drug cost, $/per cycle |  |  |  |
| sorafenib | 1198.003(958.403- 1,437.604) | Gamma | Local charge |
| Sunitinib | 318.517(254.813- 382.220) | Gamma | Local charge |
| Brivanib | 5880.865(4,704.692- 7,057.038) | Gamma | Local charge |
| Linifanib | 4151.082(3,320.865- 4,981.298) | Gamma | Local charge |
| Sorafenib Plus Erlotinib | 1804.493(1,443.594- 2,165.391) | Gamma | Local charge |
| Lenvatinib | 254.896(203.917- 305.875) | Gamma | Local charge |
| Donafenib | 2750.749(2,200.56- 3,300.898) | Gamma | Local charge |
| Sintilimab plus IBI305 | 3685.920(2,948.736- 4,423.104) | Gamma | Local charge |
| Atezolizumab plus Bevacizumab | 7693.527(6,154.821- 9,232.232) | Gamma | Local charge |
| Second-lline drugs | 3392.758(2,714.206- 4,071.310) | Gamma | Local charge |
| Test |  |  |  |
| Sorafenib group | 168.230(134.584-201.876) | Gamma | 27 |
| other group | 180.248(144.198-216.298) | Gamma | 27 |
